# A neuroimaging data set on problem solving in the case of the reversal error: Putamen data

**DOI:** 10.1016/j.dib.2020.106322

**Published:** 2020-09-19

**Authors:** Lara Ferrando, Noelia Ventura-Campos, Irene Epifanio

**Affiliations:** a Grup Neuropsicologia i Neuroimatge Funcional, Universitat Jaume I, Spain; b Dept. Educació i Didàctiques Específiques, Universitat Jaume I, Spain; c Dept. Matemàtiques-IF, Universitat Jaume I, Spain

**Keywords:** MRI, Neuroeducation, 3D shape, Algebra problem solving, Reversal error

## Abstract

Structural Magnetic Resonance Images (sMRI) for a sample of university students were recorded. Out of magnetic resonance, students performed a test of algebra problem solving. As we are interested in reversal errors, the test was prepared to detect this kind of error.

Depending on the number of mistakes made, students were divided into two groups: one group contains 15 students that responded erroneously to more than 60% of the 16 questions, and the other group contains 18 students that did not make any mistake.

We are interested in the more relevant brain structures for this neuroeducation problem. The analysis of these data can be found in Ferrando et al. (2020) [1]. The results of the volumetric analysis showed differences between groups in the right and left putamen. Therefore, both putamens were pre-processed and segmented to use them in the shape analysis.

The dataset contains the slices of the left and right putamen and the left putamen of each of 33 subjects, 20 females. It also contains a vector that indicates the group to each subject belongs to.

## Specifications Table

**Table:** 

Subject	Neuroscience
Specific subject area	Neuroimaging, education and 3D shape analysis
Type of data	Octave or MatLab file; Rdata file
How data were acquired	A 3 Tesla Philips scanner and 1.5 Tesla Siemens Symphony scanner were used to obtain the images. After, SPM12 (toolbox of MatLab) was used to analyze the images. The images were preprocessing and segmented using the method Voxel Based Morphometry (CAT12).
Data format	Raw
Parameters for data collection	Philips scanner: High-resolution T1-weighted, TR = 8.4 ms, TE = 3.8 ms, matrix size = 320 × 320 × 250 and voxel size = 0.75 × 0.5 × 0.8 mm Siemens Symphony scanner: High-resolution T1-weighted, TR = 2200 ms, TE = 3 ms, flip angle = 90°, matrix size = 256 × 256 × 160 and voxel size = 1 × 1 × 1 mm. Acquisitions covered the entire brain and were performed in parallel to the anterior commissure-posterior commissure plane (AC-PC).
Description of data collection	The slices of the left and right putamens of thirty-three participants (20 females) were collected. Besides a vector indicating the label for each subject (1 for RE-makers and 2 for non-RE makers). The ages of the participants ranges from 18 to 26 years.
Data source location	Institution: Universitat Jaume I City/Town/Region: Castellón Country: Spain
Data accessibility	With the article
Related research article	L. Ferrando, N. Ventura-Campos, I. Epifanio. Detecting and visualizing differences in brain structures with SPHARM and functional data analysis, Neuroimage [1].

## Value of the Data

• This is data set about putamen surfaces to the phenomenon of reversal error in the algebra problem solving.
• Data come from a real and important neuroeducational problem like algebra problem solving.
• Data set is useful for reproducibility and to further studies about reversal error problem.
• The data set can be used to benchmark and to compare methods of classification of 3D shapes in general.
• The data set can be beneficial to obtain a big data about the MRI segmentation of putamen.
• The data set can be used to perform a meta-analysis on the association of putamen and mathematical learning.

## 1 Data Description

**Fig. 1:**
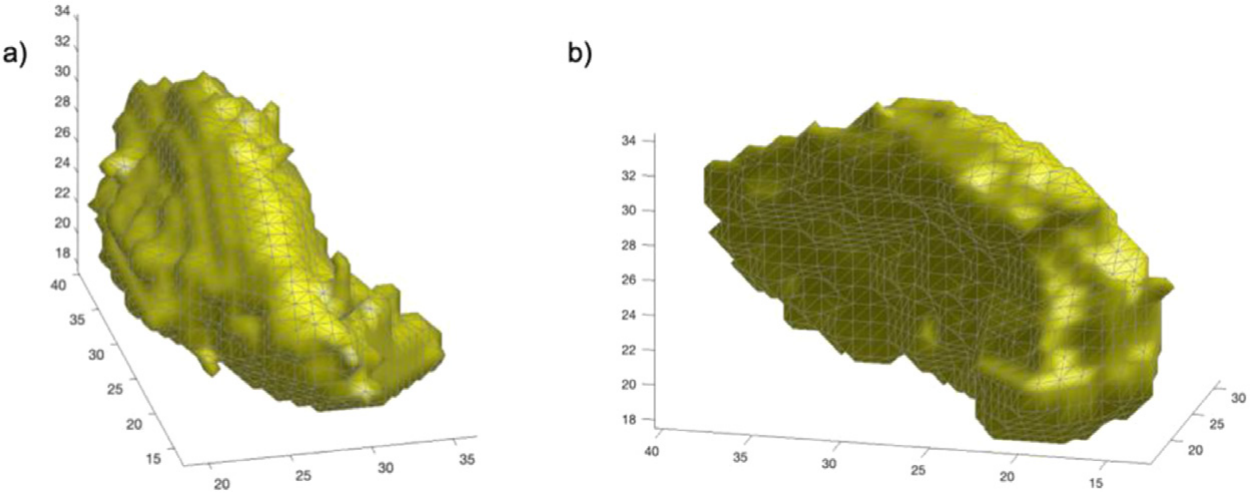
**Representation of the segmentation of Putamen.** Example of reconstruction 3D of one participant of slices of a) left putamen and b) right putamen.

The free and open Octave or MatLab file contains three variables: Hleft is a struct MatLab object with the slices of the left putamen for each participant. Hrigh is a struct MatLab object with the slices of the right putamen for each participant (see Fig. 1). The vector g contains the labels that indicates to which group each participant belongs to (1 for RE-makers and 2 for non-RE makers). Data is also provided in Rdata format for the free and open R software, with the same structure.

## 2 Experimental Design, Materials and Methods

### 2.1 Participants

In this study were collected data from thirty-three participants (20 females) with 18–26 years (mean age: 22.03, SD: 2.36). The subjects did not have any severe neurological and medical disease, traumatism, loss of consciousness, and the typical exclusion criteria when the magnetic resonance is performing.

### 2.2 Acquisition images

For the acquisition images were used two scanners. The first Philips scanner (High-resolution T1-weighted, TR = 8.4 ms, TE = 3.8 ms, matrix size = 320 × 320 × 250 and voxel size = 0.75 × 0.5 × 0.8 mm.) and the second Siemens Symphony scanner (High-resolution T1-weighted, TR = 2200 ms, TE = 3 ms, flip angle = 90°, matrix size = 256 × 256 × 160 and voxel size = 1 × 1 × 1 mm). The scanner acquisitions were performed in parallel to the anterior commissure-posterior commissure plane (AC-PC). The MRI scans were acquired while subjects were in rest.

### 2.3 Data preprocessing

The pre-processing of the images was carried out with SPM (SPM12 (v7219), Wellcome Trust Centre for Neuroimaging, London, UK, http://www.fil.ion.ucl.ac.uk/spm/software/spm12), using the methodology VBM with CAT12 toolbox to perform the pre-processing steps (CAT12.5, http://dbm.neuro.uni-jena.de/cat/). The standard pre-processing suggested in CAT12 manual was performed, where we used an 8-mm full- width-half-maximum Gaussian smoothing.

To segment the putamens, the *imcalc* toolbox of SPM12 was used together with an intersection between the image of each of the subjects with the putamen of the AAL atlas. To obtain bit map formats, MRIcro was used, so that we can get the putamen axial- slices in 2D.

## Ethics Statement

All participants were students of Universitat Jaume I. Before participating, they signed a written consent form. All experimental procedures followed the guidelines of the research ethics committee at Universitat Jaume I.

## Declaration of Competing Interest

The authors declare that they have no known competing financial interests or personal relationships which have, or could be perceived to have, influenced the work reported in this article.

## Appendix Supplementary materials

Image, application 1

## References

[1] Ferrando L., Ventura-Campos N., Epifanio I. (2020). Detecting and visualizing differences in brain structures with SPHARM and functional data analysis. Neuroimage.

